# Static and active tactile perception and touch anisotropy: aging and gender effect

**DOI:** 10.1038/s41598-018-32724-4

**Published:** 2018-09-24

**Authors:** A. Abdouni, G. Moreau, R. Vargiolu, H. Zahouani

**Affiliations:** Univ. Lyon, Laboratoire de Tribologie et Dynamique des Systèmes-LTDS UMR-CNRS 5513, ECL-ENISE, F-69134 Ecully, France

## Abstract

Although the human finger is the interface used for the touch process, very few studies have used its properties to provide a description of tactile perception regarding age and gender effects. Age and gender effects on the biophysical properties of the human finger were the main topics of our previous study. Correlating tactile perception with each parameter proved very complex. We expand on that work to assess the static and dynamic touch in addition to the touch gestures. We also investigate the age and gender effects on tactile perception by studying the finger size and the real contact area (static and dynamic) of forty human fingers of different ages and gender. The size of the finger and the real contact area (static and dynamic) define the density of the mechanoreceptors. This density is an image of the number of mechanoreceptors solicited and therefore of tactile perception (static and dynamic). In addition, the touch gestures used to perceive an object’s properties differ among people. Therefore, we seek to comprehend the tactile perception of different touch gestures due to the anisotropy of mechanical properties, and we study two different directions (top to bottom and left to right).

## Introduction

The human hand is one of the most complex structures of the human body in terms of both sensory acquisition and motor control. The anatomy of human finger is quite unique and includes the configuration of bones, nail, fingerprint and no muscles. In addition, the finger is one of the most sensitive organs of the human body as it has a very high density of receptors^1^. The latter are responsible for converting deformation stimuli such as vibration, pressure, and stretching into an electrical signal transmitted to the brain via the central nervous system^2,3^. The mechanoreceptors and the associated afferents (nerves) are the fundamental units of the nervous system for the transduction and transmission of tactile feedback to the brain. Each of these parameters affects tactile perception in addition to others such as age and gender^4–12^. Various results and hypotheses have been proposed in the literature on tactile perception and especially regarding the age effect. The studies in the literature agree on the weakening of tactile perception with age. This fact was studied from different angles such as the central nervous system^6,10^, mechanoreceptors, stimulus response rate^13–15^ and the physical properties of the finger^16^. In addition, the natural difference between men and women due to their intrinsic characteristics was also investigated^17–19^.

Tactile acuity at the back was assessed using two-point discrimination threshold in vertical and horizontal directions. Women in general show better tactile acuity than men^20^. These results have been subject to various explanations and hypotheses in the literature. One of the most convincing explanations is based on the physical difference between men and women^20^. The authors showed that tactile acuity improves with decreasing finger size, and this correlation fully explains the better perception of women, who on average have smaller fingers than men^20^. Indeed, when gender and finger size are both considered in statistical analyses, tactile acuity can only be predicted by finger size^20^. Thus, a man and a woman with fingers of equal size will, on average, enjoy equal tactile acuity^20^. Tactile perception or active touch is the ability to detect small changes in stimulus amplitude during movement^21–23^. Tactile spatial acuity is the ability to feel the difference between two closed points in static conditions^10,20,24^. Both types of touch are influenced by the distribution of the types of mechanoreceptor in the skin. The inverse relationship between tactile acuity and finger size highlights the importance of afferent density in mediating tactile spatial acuity. Estimates of afferent density in the glabrous skin on the hand have been made, based on data from single unit recordings and histological analyses of the median nerve^20,25^. Several results and explanations can be found in the literature relating to age and the gender effects on tactile perception and they do not always agree, but there are two main facts on which all the authors concur: (1) tactile perception weakens with age, and (2) women in general have better tactile spatial acuity. However, the evolution of the gender effect with age on tactile spatial acuity and perception has not been shown^5,26–29^.

Neurophysiologists have revealed the specialization of the structure and function of these receptor systems and demonstrated that each receptor system is specialized in conveying a particular type of information. Merkel cells are fired for light touch and shock stimulus^30,31^; Meissner afferents are sensitive to vibrations and pressure stimulus at low frequency^32–34^. Ruffini corpuscles are sensitive to skin stretch stimulus^34^; and Pacinian afferents are sensitive to vibrations and pressure stimulus at high frequency^34,35^. Therefore, tactile perception (dynamic contact) is more dependent on Pacinian and Meissner corpuscles. However, tactile spatial acuity (static contact) is more dependent on Ruffini corpuscles and Merkel disks. The location and the distributions of mechanoreceptors in the finger pulp give different weights to each of these organs. In addition, the mechanoreceptors such as Merkel disks and Meissner corpuscles closest to the skin surface are more numerous. Therefore, the gender effect on tactile spatial acuity can be explained via the density of Merkel disks^20^.

For tactile spatial acuity, we started by agreeing with the hypothesis that said “If Merkel cells, like Meissner corpuscles, are more densely packed in smaller fingers, then presumably the fingers of women would be endowed with greater spatial resolving power than those of men”^20^. For tactile perception, we knew that the density of Meissner corpuscles is higher in the small finger^36^. In this study, we seek the age and the gender effect on static and active tactile perception via the contact area for the flowing reasons:

- The real contact area synthesizes all the biophysical properties (contact, mechanical and topographical properties) of the human finger^37^.
- The contact area could be correlated to the number of mechanoreceptors involved, and to static and active tactile perception.
- The contact area could explain the evolution of the gender effect on static and active tactile perception with age.

In our previous study, we investigated ageing and gender influences on the biophysical properties of the human finger^37^. The results obtained showed significant differences in finger mechanical properties, contact properties and surface topography. In addition, the results demonstrated clear anisotropy of mechanical properties. In this study, we address the role played by the anisotropy of finger mechanical properties in tactile perception. In addition, the effects of the anisotropy of mechanical properties on touch gestures and tactile perception are investigated.

## Materials and Methods

Concerning the use of density of mechanoreceptors as an image of static and active tactile perception, we are based on the literature:

Merkel disks are difficult to visualize anatomically^38^, and their density with respect to finger size is unknown. However, Meissner corpuscles are more densely distributed in smaller fingers^36^. Indeed, homologous fingers in different individuals probably have the same number of Meissner corpuscles^36^.

Why does finger size affect static tactile perception? The high density of Meissner corpuscles in small fingers^36^ presumably does not affect static tactile perception, because Meissner corpuscles activate rapidly adapting Type-I (RA1) afferents that interfere with fine spatial perception (active tactile perception)^39^. In contrast, a high density of Merkel cells could improve spatial acuity (static tactile perception).

Touch receptors are not distributed evenly over the body. The fingertips and tongue may have as many as 100 per cm². However, their high density in a small area such the fingertip makes our hypothesis very strong. Therefore, we consider that the mechanoreceptors distributed evenly in the finger. Moreover, The Pacinian and Ruffini corpuscles are approximately evenly distributed over the glabrous skin area^25^.

**Figure 1:**
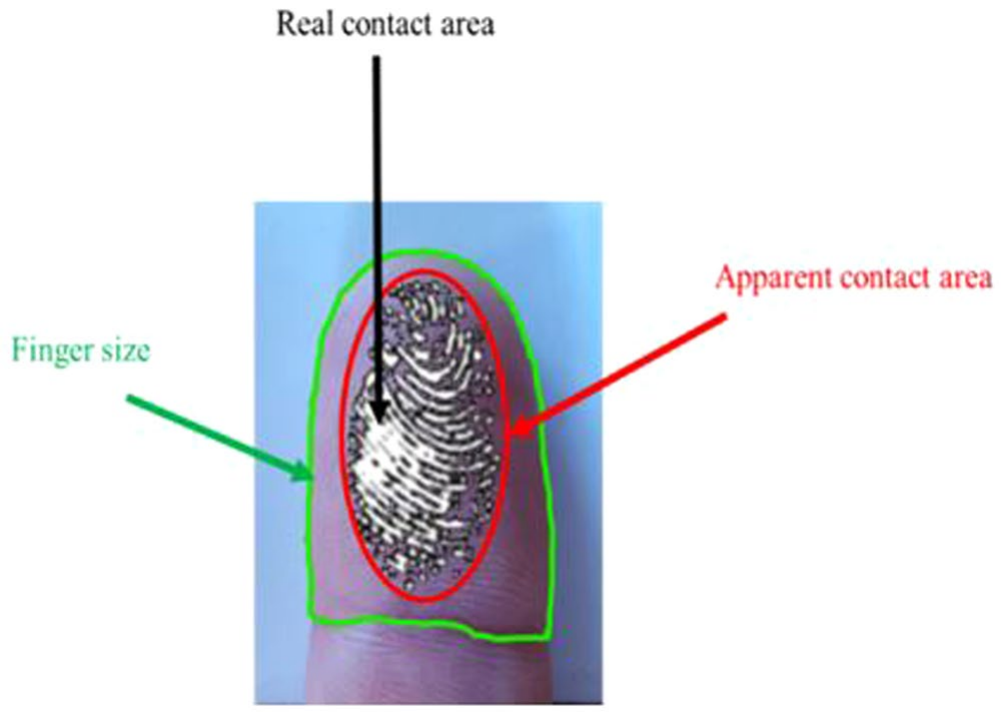
Illustration of the areas measured (finger size (green), apparent (red) and real contact areas (black)).

The contact area is the parameter that defines the number of mechanoreceptors solicited during touch. However, two types of contact area should be taken into consideration: firstly, the apparent contact area is the global contact area between the finger and the surface touched (see Fig. 1). This contact area is investigated because it can be compared to the results in the literature and linked to the biophysical properties of the human finger. Secondly, the real contact area is the contact between the fingerprint relief and the surface, as the fingerprint is not flat. Finger size is another parameter important for tactile perception and is taken into consideration.

New techniques have been developed to understand age and gender effects on the contact area. A trained sensory handfeel panel of forty French volunteers (20 women and 20 men) participated in the experiments. All the subjects were white-collar workers and all the measurements were performed *in vivo* and were non-invasive. The participants were the same as in the previous study^37^. The database was divided into four age groups (26 ± 3, 35 ± 3, 45 ± 2, 58 ± 6) of five persons each. All the volunteers were trained to control the normal force applied and the speed of sliding their finger on a surface. They were adequately informed of the aims, the methods used, and they gave their written informed consent to the protocol.

**Figure 2:**
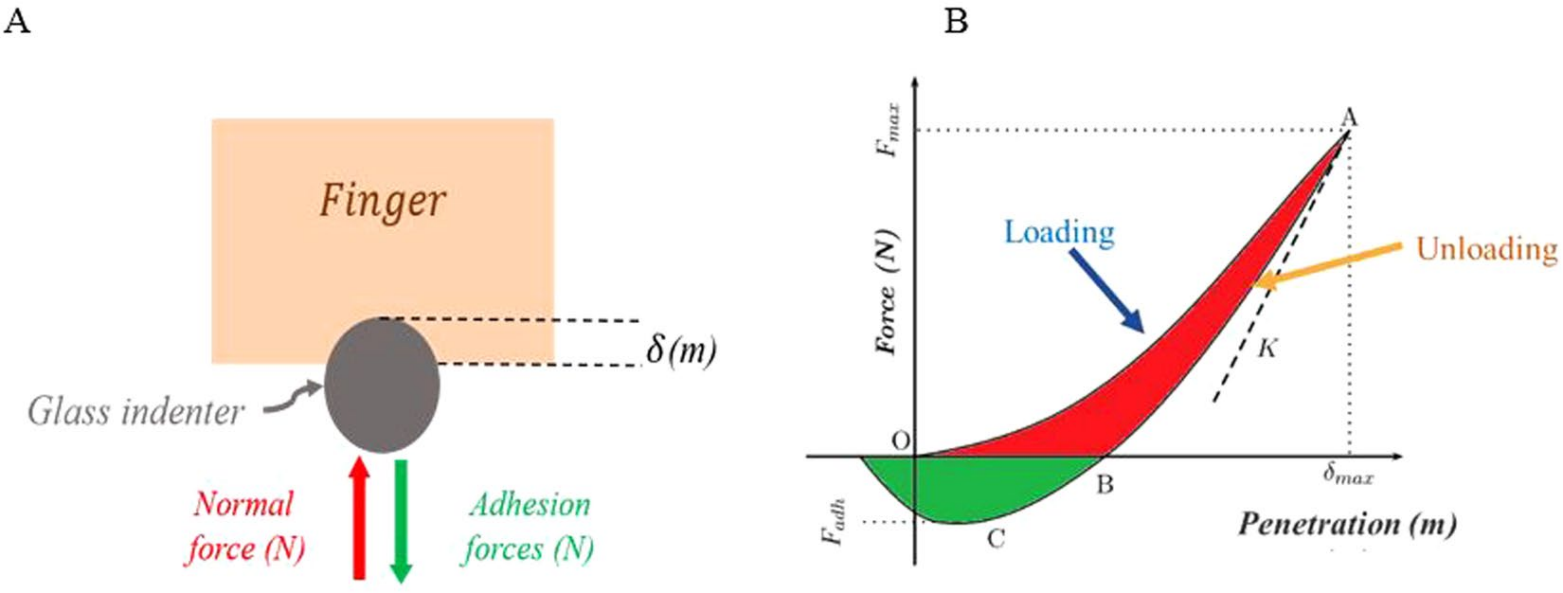
(**A**) The principle of the indentation system^37^. (**B**) Representative indentation curve on finger skin. The first part of the curve (1) corresponds to the loading phase and the second part to the unloading phase (2). The maximum adhesion force F_ad_, is the force required to break the contact between the indenter and the finger. The green area trapped by the curve corresponds to the energy of adhesion between the indenter and finger. The red area trapped between the loading and unloading phases is the energy dissipation of the material.

A new light load indentation system, based on the technique previously developed for cutaneous tissues *in vivo* and *ex-vivo*^37,40,41^, has been designed to study the finger’s real contact area properties as a function of age and gender. Indentation is the technique used most frequently for studying the mechanical properties of tissues *in vivo* or *ex-vivo*. Several *in vivo* indentation systems have been developed to facilitate the characterization of the biomechanical properties of the skin^42–45^. These include a simple loading / unloading device with an indenter. The indentation determines the properties of the material after physical contact between an indenter and the material. The load and depth of indentation are measured continuously during loading and unloading (see Fig. 2). This technique can provide much information on the properties of the materials. The mechanical characteristics of a material under compression such as contact stiffness *k*_*z*_, elasticity modulus, E, and adhesive force, can be obtained from indentation tests. These tests consist in pushing a rigid indenter perpendicularly onto the surface of a sample and recording the variation of the applied normal force, F_N_, as a function of penetration into the material, δ. Generally, the applied force is linked to the depression by a power law defined by *F*_*N*_ = *Kδ*^*n*^, where the constant K and the exponent n theoretically depend on the geometry of the indenter and the mechanical behavior of the material (see Fig. 2B). A schematic representation of the data typically obtained with an indentation system is presented in Fig. 2B.

### Contact area measurements

#### Static contact area

**Figure 3:**
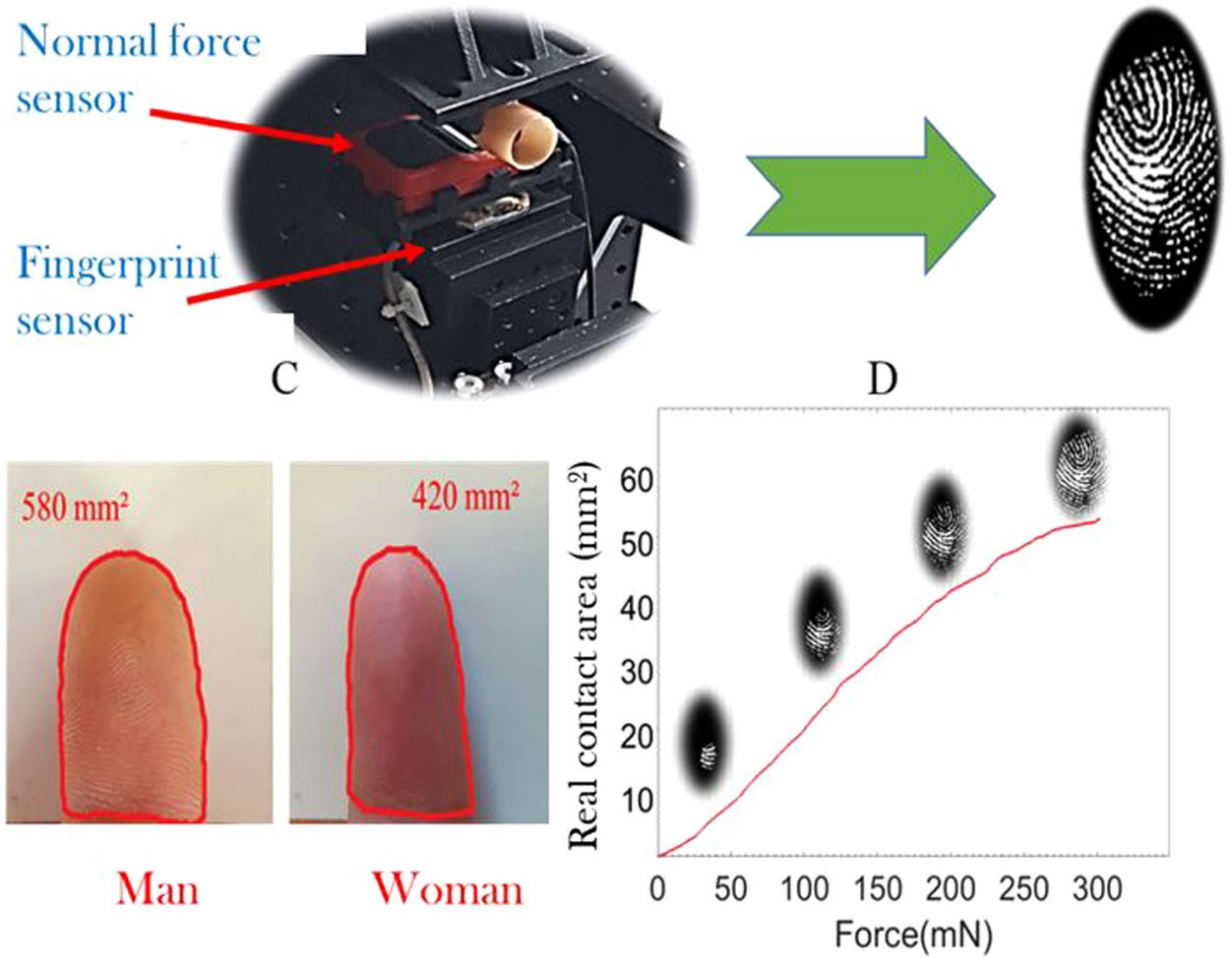
(**A**) A fingerprint sensor device coupled to an indentation system. Different sizes of plastic guides corresponding to the morphology of the fingers are used to properly position the finger in the center of the sensor reading area. (**B**) An image obtained with the fingerprint sensor at 0.3 N. (**C**) A photo of the finger and the contour of the first phalanx detected with the Canny filter. (**D**) The evolution of the contact area as a function of the force applied.

For the static real contact area study, we used a fingerprint sensor coupled to a Specific finger indentation device (see Fig. 3A,B). Different plastic finger’s guides sizes correspond to fingers morphology. They are used to well position the finger in order to indent the same predetermined area for all subjects. As already mentioned, the goal of the first part of the study is to understand static tactile perception via the static contact area, with emphasis given to age and gender effects. For our experiment, we replaced the indenter by an optical fingerprint sensor. This fingerprint reader captured videos of the fingerprint in contact as a function of the applied force with high performance sensors and a large reading surface (see Fig. 3A). For the applied force measurements, we used a force sensor with a force range up to 0.5 N and accurate to within 5 mN. After that, the size of the finger was measured with a CCD camera and a contour detection algorithm (see Fig. 3C). Firstly, a photo of the finger was captured on a white piece of paper measuring 50 * 50 mm². The contour of the first phalanx was then detected by an image processing software application (the Canny filter)^46^ (see Fig. 1). Finally, the finger size was calculated numerically with Matlab software.

The protocol used in this part can be summarized as follow. The volunteers washed their hands with tap water and Marseille soap, then wiped their hands with a paper towel and let their hands rest for 15 minutes. At the end of the rest period, the subjects placed their index finger in the plastic guide in order to position the fingerprint in the center of the reading zone of the sensor (see Fig. 3A). A computer program was used to capture a video of the fingerprint as a function of the evolution of the normal force up to 0.3 N. At the end of each measurement the subjects removed their finger and the fingerprint reader was cleaned with ethanol.

The fingerprint reader used (Morpho smart 1300) can measure the contact area with high-resolution (500 dpi, 256 gray levels) and a large reading area, i.e. 308 mm². All the measurements were carried out at constant indentation speed *V* = 0.2 mm.s^−1^ and under a maximal applied normal load F_N_ = 0.3 N.

As already mentioned in the introduction, the size of the finger defines the density of the mechanoreceptors per unit area, so that the real contact area density must be calculated with respect to the finger size. This density is an image of the number of mechanoreceptors solicited and therefore of static tactile perception. In other words, higher density means more mechanoreceptors have been solicited in the touch process, hence better tactile spatial acuity. As we focused on static tactile perception, the density was calculated with realistic parameters that corresponded to the maximum force applied (the real contact area for Fn = 0.3 N).

$${\rm{Density}}\,({\rm{numbet}}\,{\rm{of}}\,{\rm{mechanoreceptors}}/{\rm{unit}}\,{\rm{area}})=\frac{{Real}\,contact\,area}{Finger\,size}\ast 100 \% $$

#### Dynamic contact area

**Figure 4:**
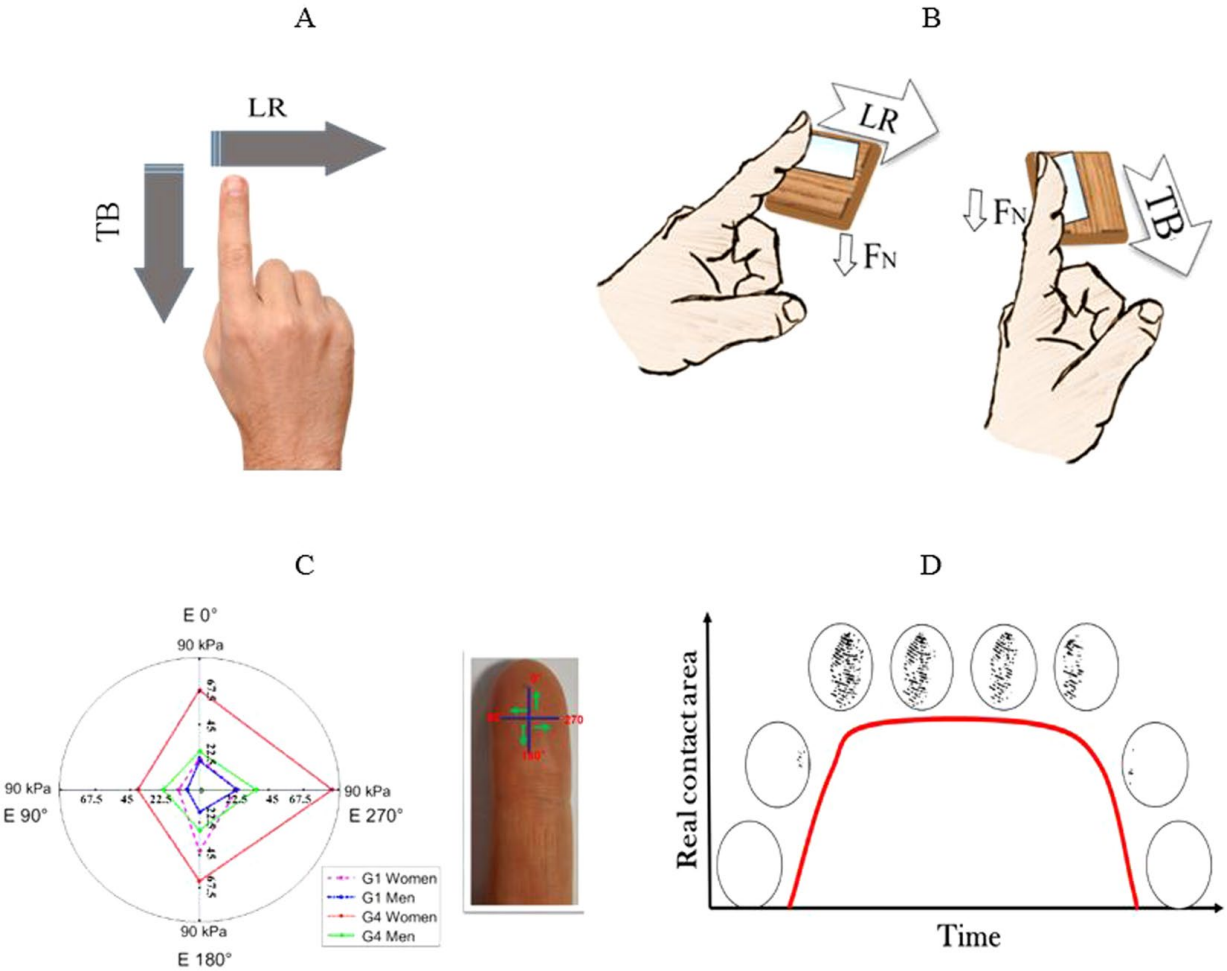
(**A,B**) The two defined directions (left to right (LR) and top to bottom (TB))^37^. 5 uniform forward translations in the two directions, normal force: 0.3–0.4 N, sliding speed: 20–30 mm.s^−1^, friction length: 10 mm. The movement is generated by the whole arm. (**C**) Illustration of the anisotropy of the finger’s mechanical properties for both men and women of the youngest and the older groups^37^. The two age groups (G1, G4) correspond to (26 ± 3 and 58 ± 6 years old), respectively, for men and women (this illustration is taken from our previous study)^37^. The evolution of Young’s modulus with age is anisotropic as a function of the direction measured. The results demonstrate a higher E for the exterior part of the finger skin (0°, 270°). The exterior part of the finger is more exposed to the environment and to repeated friction in daily life (i.e., writing); therefore, increasing anisotropy of mechanical properties with age can be observed^37^. (**D**) Illustration of the evolution of the real contact area as a function of time during the touch process.

**Figure 5:**
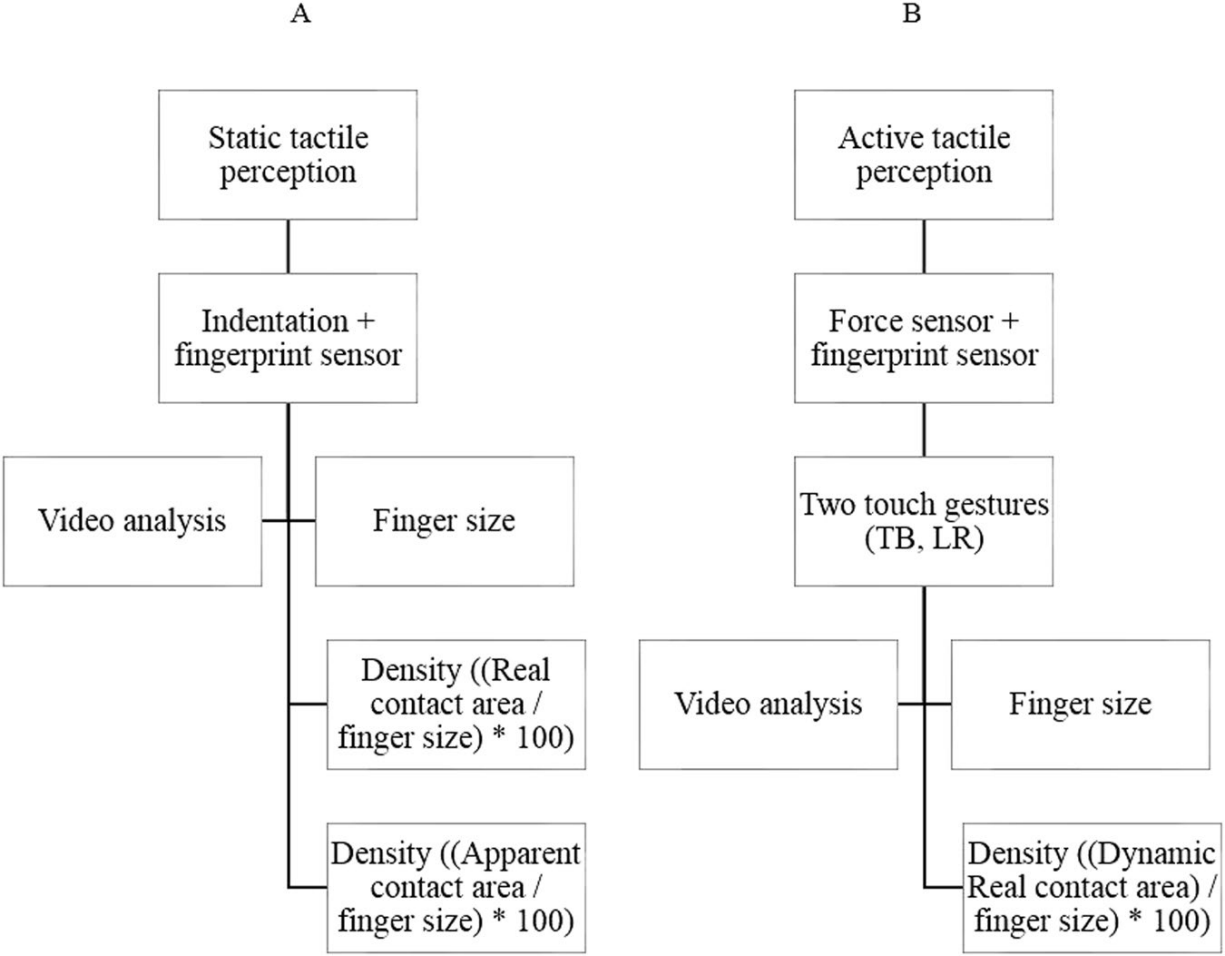
Diagram of the protocol used. (**A**) Static contact, (**B**) dynamic contact.

Clear anisotropy of the mechanical properties of human finger skin has been demonstrated in the literature^37^. Using a contactless indentation system the finger’s mechanical properties were measured in four different directions (see Fig. 4). The fact that we previously demonstrated that the finger’s mechanical properties are anisotropic led to asking a fundamental question: “What is the effect of anisotropic mechanical properties on tactile perception and touch gestures?” This anisotropy leads to differences in active tactile perception as a function of the touch gesture employed. The literature includes a study on the dynamic apparent contact areas in four different directions measured (D, U, P and R)^47^. The authors found different dynamic apparent contact areas in each direction, which agrees with the anisotropy of the mechanical properties of finger skin. We investigate this problem here. Firstly, we measure the dynamic real contact area in two different directions (top to bottom and left to right) on forty volunteers. Then, we calculate the percentage of density to understand the effects age and gender on active tactile perception. Finally, we compare the results of each touch gesture, in order to explain its effect (see Fig. 5).

For the dynamic contact area, we used the same fingerprint sensor as in the first part coupled with normal force sensor with a force range up to 0.5 N, accurate to within 5 mN. After that, all the volunteers were monitored while performing the same experimental procedure (see Figs 4 and 5). First, the volunteers washed their hands with tap water and Marseille soap, then wiped their hands with a paper towel and kept them still for 15 min in a room at a temperature of 23 ± 1 °C and 55% relative humidity (RH). Afterwards, the touch experiments were conducted in the same room. The experimental conditions used with the human finger are summarized as follows: 5 uniform forward translations in the lateral (left to right (LR)) and then in the longitudinal (top to bottom (TB)) directions (see Fig. 5), normal force: 0.3–0.4 N, sliding speed: 20–30 mm.s^−1^, friction length: 10 mm. These conditions are frequently observed in the literature and correspond to classical human handling conditions^48,49^. Finally, we calculated the percentage density of the dynamic real contact area in each direction. These densities can explain the age and gender effects on active tactile perception. In addition, we explain the effect of the anisotropy of touch gestures on active tactile perception due to the mechanical properties.

### Statistical analyses

Matlab software was used for statistical data analysis. Analysis of variance (ANOVA) is a statistical model used to analyze the differences between and among groups and to determine if the differences between the means are statistically significant. The data are considered statistically significant if the *p*-value is lower than the significance level defined (0.05 in our case). In our study, we used ANOVA to see whether we had statistically significant differences as a function of age and gender (*).

In statistics, the Pearson correlation coefficient (*r*) is a measure of the linear dependence (correlation) between variables. The Pearson correlation coefficient value was used to determine the strength of the relationship between variables. The correlation is considered strong for an r value higher than 0.8^50^.

## Results

**Figure 6:**
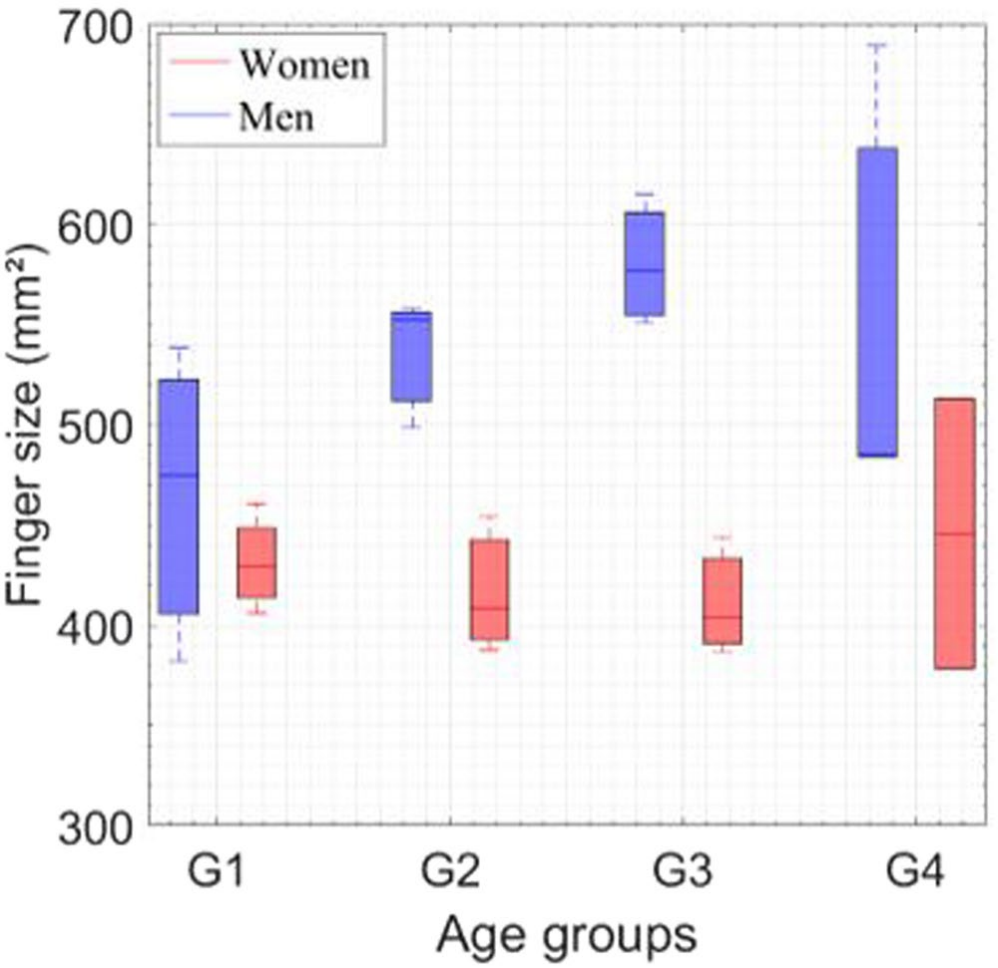
Finger size as function of age groups for men and women. Correlation result between finger size and age groups for men and women is illustrated. The four age groups (G1, G2, G3, and G4) correspond to (26 ± 3, 35 ± 3, 45 ± 2, and 58 ± 6 years old), respectively, for men and women. Pearson’s statistical analyses: (+ for r > 0.8, − for r < −0.8) highly correlated, (0) for no correlation, R indicates the correlation results, ^*^p < 0.05 for statistically significant. The statistics analysis shows significantly larger finger size for men for all age groups (p < 0.05).

Figure 6 shows the age and gender effects on finger size. Finger size is significantly larger for men for all age groups (p < 0.05). Although finger size is an individual property of the finger, a positive correlation with age for men can be seen. For women, finger size is negatively correlated to age. In our previous work, we explained the topographic differences between men and women by the effect of callosity^37^. This phenomenon increases the thickness of the *stratum cornum* layer only for men. This hypothesis could explain the results of finger size with age^37^. In addition, we explained why women are less affected by callosity due to cosmetic products. In addition, they have higher water loss, which could affect finger size by decreasing the thickness and size of skin layers.

### Apparent and real contact areas: age and gender effects

**Figure 7:**
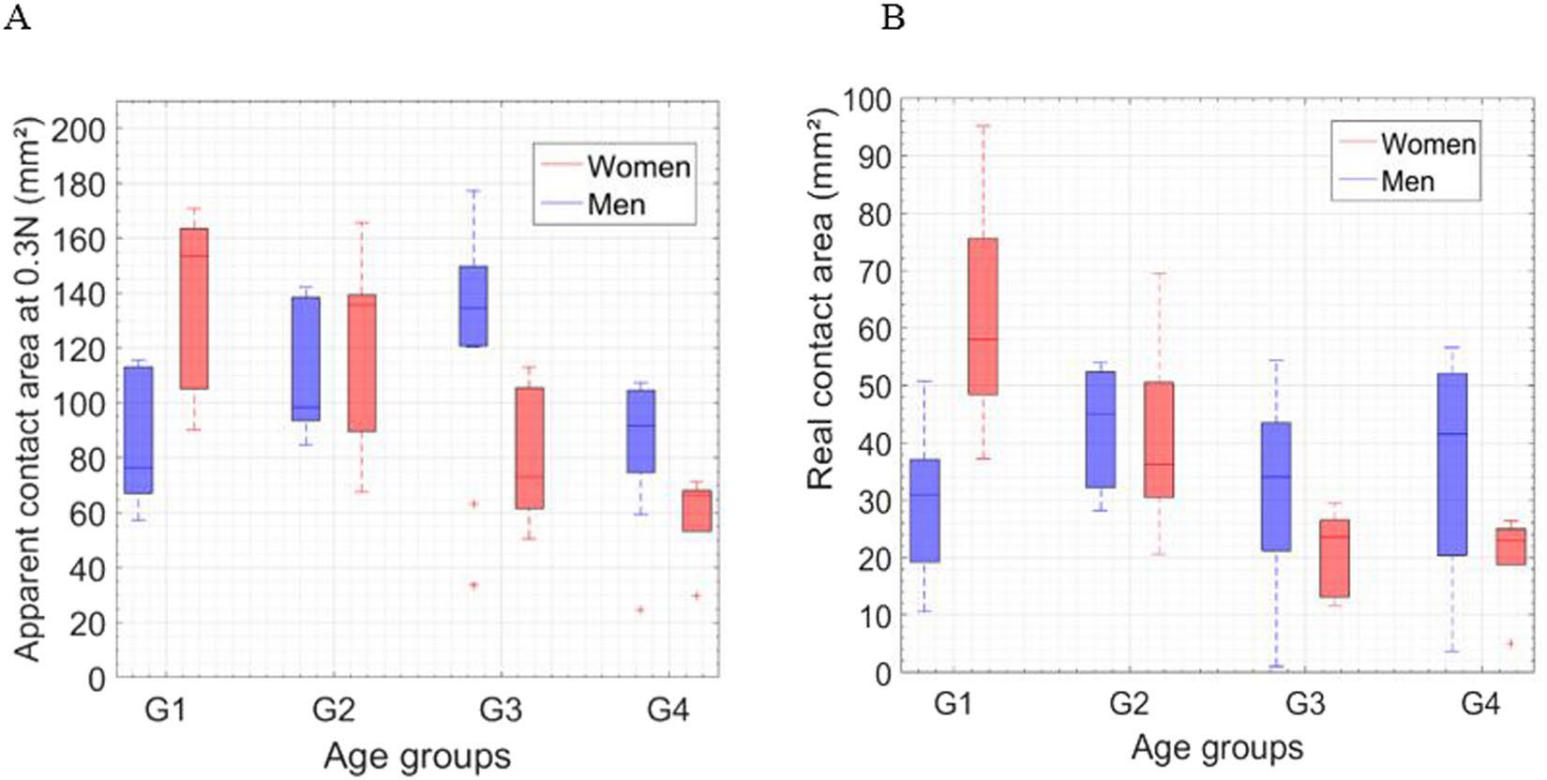
(**A,B**) The apparent and real contact areas as a function of age and gender respectively, captured on video. The four age groups (G1, G2, G3, and G4) correspond to (26 ± 3, 35 ± 3, 45 ± 2, and 58 ± 6 years old), respectively, for men and women. Pearson’s statistical analyses: (+ for r > 0.8, − for r < −0.8) highly correlated, (0) for no correlation, R indicates the correlation results, ^*^p < 0.05 for statistically significant.

Figure 7A highlights the apparent contact area of the finger at 0.3 N as a function of age group. The apparent contact area clearly reduces with age for women (see Fig. 7A). This reduction is negatively correlated to age. These results are in line with our previous work where we calculated the apparent contact area based on the JKR theorem^37^. The results obtained for women can be explained simply by the high increase of their finger skin’s Young’s modulus, as described in the literature^37^. On the contrary, the apparent contact area for men is not linearly correlated to age. These results can be explained by finger size and by the lower influence of age on their mechanical properties^37^ (see Fig. 7A,B).

The women of the first age group have a significantly larger apparent contact area (p < 0.05), after which the gender effect is inversed after the age of 40. The effects of age and gender on the real contact area are very similar to those of the apparent contact area (see Fig. 7B). However, the ratio of the real contact area over the apparent contact area is higher for women, which is correlated to the fact that women have higher density fingerprint reliefs (see Fig. 7). This phenomenon has already been demonstrated with *SMa* (multi-scale arithmetic mean of roughness amplitude) and in the literature^19,28,36,37,47,51–53^.

### Density vs. static tactile perception: age and gender effects

**Figure 8:**
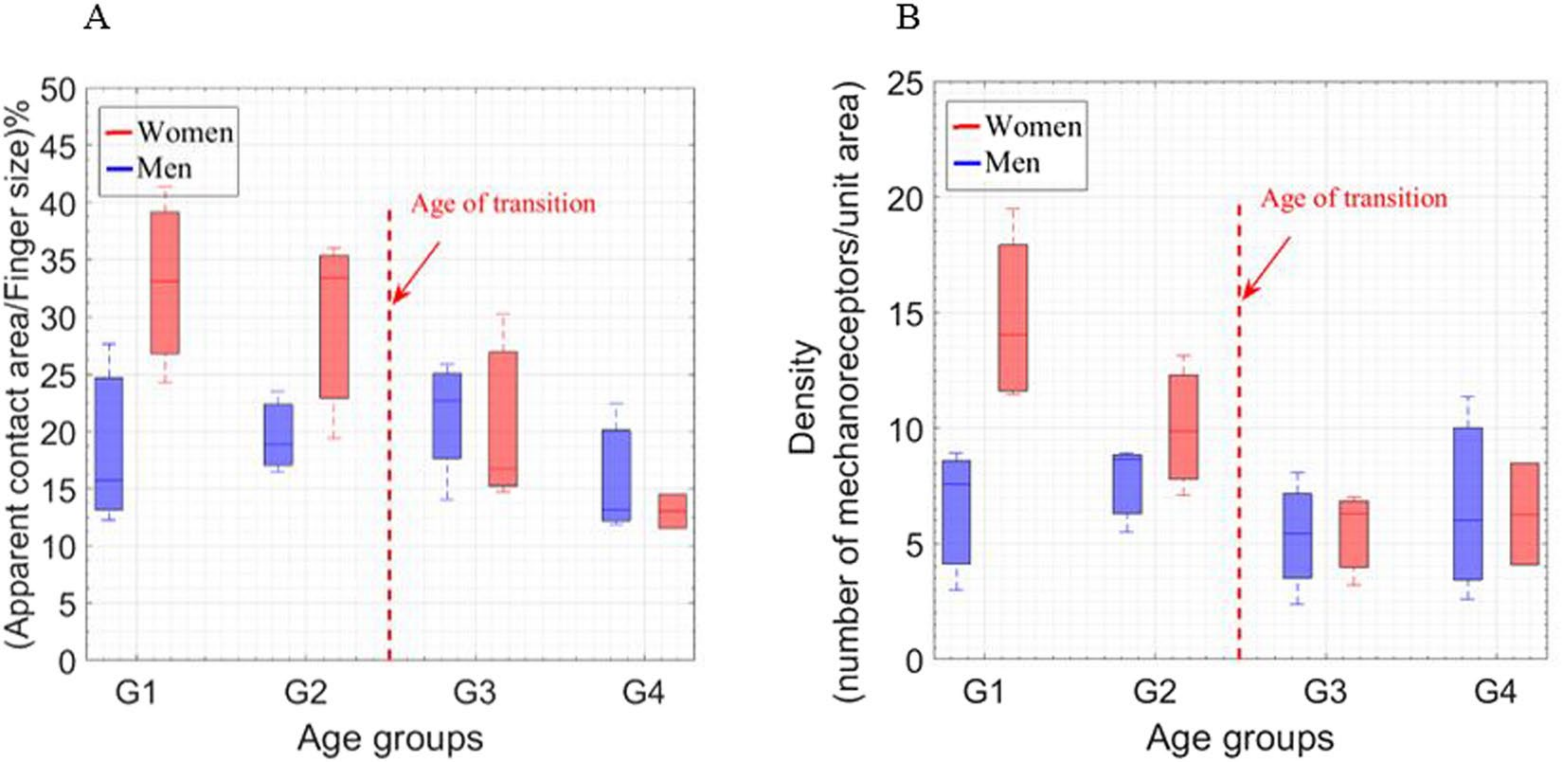
(**A**) Ratio of apparent contact area over finger size. (**B**) Density of real contact areas as a function of age and gender, via the captured video. The four age groups (G1, G2, G3, and G4) correspond to (26 ± 3, 35 ± 3, 45 ± 2, and 58 ± 6 years old), respectively, for men and women. Pearson’s statistical analyses: (+ for r > 0.8, − for r < −0.8) highly correlated, (0) for no correlation, R indicates the correlation results, ^*^p < 0.05 for statistically significant. The statistics analysis shows significantly larger finger size for women for G1 and G2 age groups (p < 0.05). Then, the density results are almost the same for men and women.

To obtain more conclusive results comparable to the literature, we calculated the ratio of the apparent contact area over finger size (see Fig. 8A). This percentage is correlated to the rate of deformation, which clearly decreases more for women with age due to mechanical properties^37^. Age and gender effects on the apparent contact area over finger size are in full agreement with the mechanical properties of the human finger^37^. Regarding the density of the real contact area, a decrease in density can be correlated to the weakening of static tactile perception with age (see Fig. 8B). The difference between men and women can be divided into two main parts. Firstly, up to the age of 40 women have a significantly higher percentage density (p < 0.05) which means higher static tactile perception according to our hypothesis. Secondly, the density results are almost the same for men and women. We interpreted this as giving the same static tactile perception. Our hypothesis was that “static tactile perception is better for women until the age of transition, after which it is almost the same”. This hypothesis is supported by the high increase in the mechanical properties of women as a function of age^37^, leading to a greater loss of skin deformation capacity, in turn leading to a loss of static tactile perception. In addition, if we take all the volunteers as one age group, women have higher contact area density and tactile spatial acuity, which is in agreement with the literature^20^.

In conclusion, according to our hypothesis the static tactile perception is inversely correlated to finger size, and positively correlated (r > 0.8) to the density of the real contact area. The real contact area and the biophysical properties of the human finger can be used to explain age and gender effects on static tactile perception.

### Density of dynamic real contact area vs. active tactile perception and touch gestures: age and gender effects

**Figure 9:**
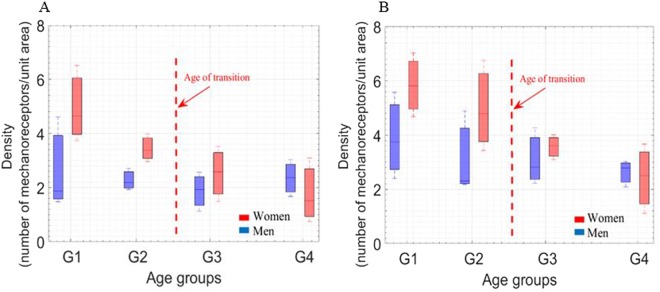
(**A,B**) Density of real contact area for (TB and LR) directions as a function of age and gender, respectively. The four age groups (G1, G2, G3, and G4) correspond to (26 ± 3, 35 ± 3, 45 ± 2, and 58 ± 6 years old), respectively, for men and women. Pearson’s statistical analyses: (+ for r > 0.8, − for r < −0.8) highly correlated, (0) for no correlation, R indicates the correlation results, ^*^p < 0.05 for statistically significant. The statistics analysis shows significantly larger finger size for women for G1 and G2 age groups (p < 0.05). Then, the density results are almost the same for men and women. The left to right touch gestures show higher density for all age groups.

Figure 9 shows the density of dynamic real contact area as function of age and gender. The anisotropy of density as a function of the touch gesture directions measured (LR and TB) is clear.

Age effect: for both men and women the percentage density decreases with age for all directions (LR and TB). The tendency is different between directions. This can be explained by the anisotropy of mechanical properties (higher Young’s modulus in the TB direction). The decrement of percentage density is in accordance with the fact that tactile perception weakens with age.

Gender effect: generally, women have higher percentage densities until 40 years old, which is in accordance with the hypothesis of age of transition stated previously. The highest percentage density was observed in the left to right (LR) direction which is in accordance with the mechanical properties^37^. We hypothesize that active tactile perception differs as a function of the touch gestures employed. In view of the results obtained, we conclude that tactile perception is better in the LR direction. These findings are in accordance with our previous results about the effect of gender and touch gestures on tactile perception^54^.

In conclusion, according to our hypothesis women have better static and active tactile perception until the age of transition, after which they are almost the same as those of men. This hypothesis is valid for two different directions (TB and LR). In our daily life, we use different touch gestures to qualify any surface. The most useful touch gestures are the lateral (LR) and the longitudinal (TB) directions, where we generally use the lateral direction for fine surfaces and the longitudinal direction for rough surfaces^54^. The results obtained are in accordance with human behavior since the lateral direction is used for fine surfaces to ensure better tactile perception^55^.

## Summary and Conclusion

This study provided *in vivo* measurements of 40 human fingers. Static and active tactile perceptions as a function of age and gender and touch gestures were investigated via the real contact area and finger size. For static tactile perception, a new system based on replacing the indenter with a fingerprint sensor was used. The latter captured a live video of the fingerprint in contact. The video captured during the indentation test was used to calculate the real contact area. This contact area was divided by the finger size in order to correlate the results to the static tactile perception. The active tactile perception and touch gestures were studied via the dynamic real contact area and the density percentage.

Touch is a result of all the mechanoreceptors, but they differ in firing frequency, size, type of firing stimulus, location and density. Therefore, some authors have attached more importance to certain mechanoreceptors such as Merkel disks for static tactile perception^20^, and Meissner corpuscles for active tactile perception, because they are very dense (small) in the finger pulp and very close to the skin surface (shallow). It is interesting to note that the threshold of active tactile perception of vibrations is positively proportional to the increase in the density of Merkel disks and Meissner corpuscles^56^. In addition, their densities in the hand are much higher than Pacinian and Ruffini corpuscles, where Merkel disks and Meissner corpuscles are reported to account for approximately 68% of the population, with Meissner corpuscles being the most abundant^57^. Estimates vary from study to study; however Meissner corpuscles, Merkel disks, and Pacinian and Ruffini corpuscles are typically reported to account for approximately 43%, 25%, 13% and 19% of the median population respectively^1,57–59^. This is why Merkel disk and Meissner corpuscle densities have been studied to understand static and active tactile perception. For both mechanoreceptors, finger size is identified as being directly correlated to their densities in the finger skin. The percentage of real contact area over finger size is an image of the number of mechanoreceptors solicited. This percentage, i.e. density, is the only parameter capable of explaining static and active tactile perception. The density calculated from the static real contact area (video capture) was used to understand static tactile perception, and age and gender effects. In addition, the density of the dynamic contact area was used to understand active touch and tactile perception as a function of age and gender.

The results obtained gave a clear explanation of age and gender effects on static and active tactile perception. Therefore, two hypotheses were proposed: firstly, women have better static and active tactile perception until 40 years old, after which both men and women enjoy almost the same static and active tactile perception. This hypothesis is not totally in agreement with the hypothesis based only on finger size because, more than finger size, the biophysical properties of the finger should be taken into consideration in order to understand the age and gender effects on static and active tactile perception. Therefore, the real contact area and density (contact area over finger size) are more appropriate for determining static and active tactile perception as a function of age and gender. Secondly, the anisotropy of the mechanical properties of the human finger leads to the anisotropy of tactile perception^54^. This means that tactile perception differs as a function of the touch gesture used. In this work, we studied two different directions and found better active tactile perception in the lateral direction. This agrees with what has been observed in human behavior, and it is a pronounced condition that can be used to mimic human touch perception artificially. The results obtained are in accordance with our previous findings. The left to right touch presented the best tactile perception^54^.
